# Oncological Genetic Counseling in Hereditary Breast and Ovarian Cancers and Lynch Syndrome High‐Risk Subjects: Evaluation of Efficacy and Outcomes Using the Genomics Outcome Scale

**DOI:** 10.1155/humu/7754087

**Published:** 2026-03-09

**Authors:** Elena Maccaroni, Rebecca Chiariotti, Riccardo Giampieri, Francesca Bianchi, Cristiana Brugiati, Laura Belvederesi, Elisa Ambrosini, Natalia Chiodi, Emma Nicol Serritelli, Francesca Morgese, Veronica Agostinelli, Giulia Mentrasti, Cecilia Copparoni, Alice Magnarini, Chiara De Filippis, Elena Burattini, Raffaella Bracci, Rita Chiari, Michela Del Prete, Renato Bisonni, Luca Faloppi, Nicola Battelli, Rosa Rita Silva, Rossana Berardi

**Affiliations:** ^1^ Department of Oncology, Azienda Ospedaliero Universitaria delle Marche, Ancona, Italy; ^2^ Department of Oncology, Università Politecnica delle Marche, Ancona, Italy, univpm.it; ^3^ Department of Oncology, Università Politecnica delle Marche, Azienda Ospedaliero Universitaria delle Marche, Ancona, Italy, univpm.it; ^4^ Medical Oncology Unit, AST1, Pesaro, Italy; ^5^ Medical Oncology Unit, Ospedale A. Murri, Fermo, Italy; ^6^ Medical Oncology Unit, Ospedali Santa Maria Della Pietà e Bartolomeo Eustachio-AST di Macerata, Camerino, San Severino Marche, Italy; ^7^ Medical Oncology Unit, Macerata Hospital, Macerata, Italy; ^8^ Medical Oncology Unit, AST2, Fabriano, Italy

**Keywords:** genetic test, Genomics Outcome scale, hereditary breast and ovarian cancers, hereditary cancers, Lynch syndrome, oncological genetic counseling

## Abstract

**Background:**

Validated tools assessing oncological genetic counseling (OGC) quality are lacking.

**Methods:**

We assessed OCG effectiveness using italian‐translated version of the Genomics Outcome Scale (GOS) questionnaire. Clinical variables were collected and their association with different answers was assessed by Fisher′s exact test or Chi‐square test for either dichotomous or other categorical variables, respectively, with level of statistical significance *p* = 0.05.

**Results:**

Between November 2024 and February 2025, 209 subjects who received the complete OGC program at Our Center responded to the questionnaire; median age was 56 years (25–81). Most (76%) had breast cancer, 72% received a negative test, 15% positive test, and 13% noninformative test with variant of unknown significance (VUS). Most patients answered affirmatively to Question 1, focused on OGC understanding: age (*p* = 0.0181) and education (*p* = 0.0028) yielded different answers. Question 2, assessing relatives risk understanding, was answered completely/partially affirmative by 94% of subjects: test result (negative noninformative vs. positive vs. VUS) was associated (*p* = 0.0175) with different answers. To Question 3, related to concern, 65% confirmed their worry: education (*p* = 0.0392) and cancer type (*p* = 0.0128) yielded different answers. In Question 4, focused on surveillance understanding, 77% declared full or partial awareness, regardless of examined factors. In Question 5, enquiring decisional ability for themselves or family members, 72% stated they were completely/partially able to make decisions. Education (*p* = 0.0287) and genetic test result (*p* = 0.0090) yielded different answers. In Question 6, reflecting future planning, 69% responded completely/almost completely affirmatively, 17% were uncertain, and 14% responded partially/completely negatively, regardless of examined clinical factors.

**Conclusions:**

GOS questionnaire confirms that OGC is useful and effective to inform patients about their condition, surveillance, and prevention. Higher levels of empowerment were seen in younger patients and those with higher education.

## 1. Introduction

The oncological genetic counseling (OGC) and associated testing services offer useful information and clinical benefits to individuals and families affected by pathological conditions with a suspected genetic etiology [1–10].

Several studies showed that genetic counseling can lead to a greater likelihood of accurately explaining disease occurrence risk and available treatment strategies; this leads to increased patients′ knowledge and perceived personal control. All these factors contribute to positive health behaviors such as increased compliance and adherence to surveillance protocols and decrease in anxiety, worry, and decisional conflict [11–13].

To date, both in Italy and in European countries, within oncology genetics services validated assessment tools aiming at evaluating the impact of received information on patients′ health‐related quality of life (HRQoL) during the OGC process are not routinely used in clinical practice. In fact, even though OGC′s main objective is to increase life expectancy and improve quality of life in families harboring different genetic conditions, estimation of benefit could be more difficult compared to other medical conditions. Indeed, even though during OGC prophylactic pharmacological and surgical options may be discussed, measurement of the beneficial impact of patients′ perceived need to undergo such procedures is rarely assessed [14, 15].

However, HRQoL measures have been widely recognized as useful tools in clinical trials, leading to their application also in the field of medical genetics [16]. HRQoL is measured using patient‐reported outcome measures (PROMs). PROMs are assessed by short, self‐administered questionnaires that capture some aspects of patients′ health status or HRQoL that are directly reported from patients, thus avoiding answers interpretation by a physician [17]. Generic HRQoL measures are available but there is no specific HRQoL PROM developed for genetic conditions that would highlight all the relevant patients′ benefits [18, 19].

For this purpose, in 2011, a quality assessment tool called “Genetic Counseling Outcome Scale” (GCOS‐24) was developed by McAllister et al., aiming at providing a PROM specific to clinical genetics services [20]. The GCOS‐24 was based on the concept of patient “empowerment”, which includes five subdimensions in which the various outcomes are summarized: cognitive, decisional and behavioral control, emotional regulation, and hope. This tool has been proven to be valid, reliable, and applicable and has been used to evaluate and improve quality in genetic counseling services. The authors have reported results of psychometric tests suggesting that GCOS‐24 might be used as PROM to evaluate clinical genetics services, although future validation studies are needed. Subsequent studies have indeed confirmed GCOS‐24 clinical utility as a tool for proper assessment of genetic counseling services effectiveness [21, 22].

Although the GCOS‐24 is currently one of the most widely used instruments for assessing PROMs in medical genetics services and genetic counseling clinics, GCOS‐24′s main limitation is its complexity; GCOS‐24 is composed of 24 items, each with seven possible answers. Moreover, GCOS‐24 generates an overall “empowerment” score, but the exact role that can be attributed to different scores among the various items is not understood. Further studies are needed to attribute “preference weights” to the various measures obtained, which reflect the value or priority attributed to each item by the target population [23]. This would clarify what interpretation can be attributed to the variations in scores.

A shorter version of the scale might be focused on proper assessment of such “preference weights”, thus facilitating its future use in place of the standard GCOS‐24.

In recent years, genetic testing has been increasingly performed outside of traditional service provision models within genetics services and clinics and will increasingly be applied to other specialties in the future. This process is called “mainstreaming genetic testing” and is precisely what is occurring in the context of cancer predisposition genes [24].

On this basis, there is a rising need to have a valid and reliable PROM that could be used to evaluate genetic and genomic counseling and testing both within and outside of clinical genetics services. An additional benefit of a shorter test would be to reduce completion times, which could also facilitate integration into clinical care and also patients′ compliance to complete such questionnaire.

To this end, in 2019, Grant et al. published the results of a study whose aim was to evaluate the effectiveness of a simplified version of the GCOS‐24 questionnaire. The outcome was the development of a shortened (six‐item) version of the GCOS, called the Genomics Outcome Scale (GOS) (Table 1).

**Table 1:** Genomics Outcome Scale (GOS) [25].

Genomics Outcome Scale	Strongly disagree	Disagree	Neither agree nor disagree	Agree	Disagree
I can explain what the condition means to people outside family who may need to know	**1**	**2**	**3**	**4**	**5**
I know who else in my family might be at risk for this condition	**1**	**2**	**3**	**4**	**5**
When I think about the condition in my family, I get upset	**1**	**2**	**3**	**4**	**5**
I know what I can do to change how this condition affects me/my children	**1**	**2**	**3**	**4**	**5**
I am able to make plans for the future	**1**	**2**	**3**	**4**	**5**
I can make decisions about the condition that might change my future or my child(ren)′s future	**1**	**2**	**3**	**4**	**5**

The GOS appears potentially suitable to use in clinical practice as means to evaluate genetic counseling and testing services, maintaining the ability of GCOS‐24 to capture the theoretical construct of empowerment [25].

The aim of our study was to use an Italian‐translated version of the GOS, during the activity of the Highly Specialized Regional Reference Center for Oncological Genetics “Prof. Riccardo Cellerino” of the Azienda Ospedaliero Universitaria (AOU) delle Marche (Table 2) [26], as a tool to evaluate the effectiveness of the OGC process, in order to measure its usefulness, especially the posttest phase, its effectiveness and the outcomes in terms of received information, clinical benefit and acceptance of proposed surveillance path based on test result, patients′, and their families′ awareness and psychological impact of the process. This form of assessment might also be useful in order to highlight any faulty areas of OGC that should be corrected in order to improve the overall quality of service offered.

**Table 2:** Italian‐translated and adapted version of the Genomics Outcome Scale (GOS).

Italian‐translated and adapted version of the Genomics Outcome Scale (GOS	1	2	3	4	5
	NoStrongly disagree	Più NO che SI’Disagree	In parte/Non sono sicuro/Né SI’ né NONeither agree nor disagree	Più SI’ che noAgree	SI’Strongly agree
1. ITA – Sono in grado di spiegare la mia condizione/storia/informazioni ricevute in consulenza alle persone che abbiano bisogno di conoscerle.1. ENG – I can explain what the condition means to people outside family who may need to know.					
2. ITA – Ho compreso quali altri parenti possono essere a rischio per la mia stessa condizione/storia.2. ENG – I know who else in my family might be at risk for this condition.					
3. ITA – Quando penso alla mia condizione/storia, mi preoccupo.3. ENG ‐ When I think about the condition in my family, I get upset.					
4. ITA ‐ So che cosa posso fare per modificare l’impatto della mia condizione/storia su di me o i miei parenti/figli.4. ENG – I know what I can do to change how this condition affects me/my children.					
5. ITA – Sono in grado di prendere decisioni riguardo la mia condizione/storia che potrebbero cambiare il mio futuro o quello dei miei parenti/figli.5. ENG – I can make decisions about the condition that might change my future or my child(ren)′s future.					
6. ITA – Mi sento in grado di fare progetti per il futuro.6. ENG – I am able to make plans for the future.					

Abbreviations: ITA, Italian, ENG, English.

## 2. Materials and Methods

Patients eligible for this study were all consecutive patients that, between November 2024 and February 2025, attended the Highly Specialized Regional Reference Center for Oncological Genetics “Prof. Riccardo Cellerino” of the AOU delle Marche and who received a complete OGC program between June 2023 and December 2024, including the following:

• Pretest genetic counseling
• Genetic testing for hereditary tumors (for hereditary breast/ovarian cancer [HBOC] syndrome and/or Lynch syndrome [LS]), performed with a multigene panel using NGS (next generation sequencing) technology, that analyzes the following genes: BRCA1, BRCA2, PALB2, CHEK2, TP53, ATM, RAD50, RAD51C, RAD51D, MLH1, MSH2, MSH6, PMS2, and EPCAM. The data obtained from the gene sequences were analyzed using a bioinformatics software that first identifies all the variants present in the analyzed genomic sequence and then, through a predefined filtering system, selects those that could have a meaning for the genes under examination. If the analysis highlights the presence of a variant, the investigation was then repeated and confirmed using a second method, namely direct Sanger sequencing, which allows the detected variant to be typed with absolute precision. If the NGS analysis highlighted the presence of a copy number variation, that is, a large deletion or insertion of one of the analyzed genes, a second technique was used that allows the identification of large gene rearrangements, namely the MLPA (multiplex ligation probe amplification) technique.
• Posttest genetic counseling, during which the test report was delivered and the result was explained.

Interpretation of identified variants′ pathogenic significance was performed according to ENIGMA consortium (evidence‐based network for the interpretation of germline mutant allele—https://enigmaconsortium.org/), American College of Medical Genetics and Genomics (ACMG) guidelines, and InSiGHT variant database (available at http://www.insight-group.org), and then, mutations were classified according to the five‐class system IARC system recommended by the International Agency for Research on Cancer (IARC) [27–30].

Eligible patients were contacted by telephone, and after, they were asked for their informed consent to participate. They were also informed that the answers had to be free and truthful, that they would not affect the clinical decisions, and that it would be possible to add free comments in the final section.

Patients who agreed to participate in the study were asked to fill out an adapted version of the GOS questionnaire that was translated into Italian, which is shown in Table 2.

The questionnaire could be answered either by e‐mail (by sending a link to access and filling out the questionnaire anonymously. The link to the questionnaire was created using the “Google Forms” function) [31] or by answering directly to questions by telephone.

The following patients′ clinical data were collected: age, gender, type of oncological diagnosis, and level of education (primary school diploma, lower secondary school–middle school diploma, upper secondary school–high school diploma, bachelor′s degree, master′s degree, PhD, and other education, for example, artistic or musical degree).

All patients were asked to provide an answer to the questions contained within the GOS, whose possible answers ranged from a scale of 1–5 (Table 2).

The first question measured the degree of understanding and ability to re‐elaborate the information received, as far as to be able to relate such information to other persons who might be interested.

The second question measured the depth of understanding the concept of hereditary transmission of the genetic risk found.

The third question measured the presence or absence of concern associated with the clinical implications of the test result

The fourth question measured the degree of understanding of the surveillance measures proposed for oneself and for one′s family members.

The fifth question measured the patient′s ability to make decisions for themselves and, most importantly, for their relatives.

The sixth question focused more precisely on the impact of the information acquired during genetic counseling on life planning.

Finally, the patient was asked to indicate the year in which the test was performed (2023 vs. 2024) and the type of genetic test result, for which three answers were possible: negative/noninformative result (no pathogenic variant found), positive result (pathogenic variant found), and noninformative result due to the detection of a variant of unknown significance (VUS)

The association between categorical variables was assessed by Fisher′s exact test for dichotomous variables and by the Chi‐square test for all other categorical variables. The level of statistical significance was set at 0.05. All statistical analyses were conducted using MedCalc 19.7.2 software for Windows and R software for Windows 4.4.2.

## 3. Results

### 3.1. Patient Characteristics

Globally, between November 2024 and February 2025 (data cutoff), 462 consecutive subjects, attending the Highly Specialized Regional Reference Center for Oncological Genetics “Prof. Riccardo Cellerino” of the AOU delle Marche, were considered potentially eligible patients. All patients had received a complete OGC program (pretest genetic counseling, genetic testing and posttest genetic counseling) in the period between June 2023 and December 2024. Among them, 54 (12 %) were excluded as they were already deceased at the time of the study.

The 408 patients alive at the time of the study were contacted by telephone to ask them to complete the questionnaire. Of these, 122 (30 %) did not answer the phone call, whereas 23 (6 %) received the phone call and refused to participate in the study: thus, the remaining 263 (64 %) patients received their questionnaire for completion.

**Figure 1:**
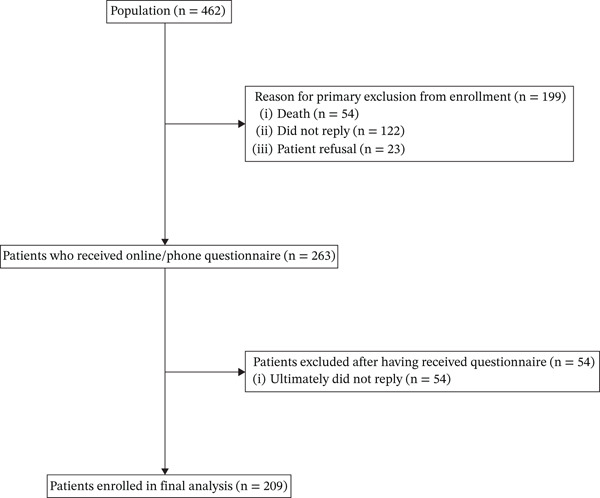
CONSORT diagram of study population.

A total of 209 (79 %) completed questionnaires were received. Figure 1 shows the CONSORT diagram relating to the disposition of the study population.

### 3.2. Distribution of Responses by Clinical Factors

In total, 209 patients responded to the questionnaire sent. Only 197 (94%) out of 209 patients responded to questions concerning their age. The median age of the subjects who responded to the questionnaire was 56 years (range 25–81). Two hundred eight (99.5%) subjects responded to the question of gender. Of these, 192 (92%) subjects were female, and 16 (8 %) patients were male.

Regarding the question on the type of tumor, 207 (99%) out of 209 subjects specified the type of tumor they were affected by. The majority of subjects (*n* = 157*%*–76*%*) who responded to the questionnaire have been diagnosed with invasive breast cancer, followed by healthy subjects (*n* = 15*%*–7*%*), patients with gastrointestinal neoplasms (*n* = 14*%*–7*%*), patients with gynecological cancer (n = 6%–3%), urological cancer (*n* = 6*%*–3*%*), and multiple neoplasms (*n* = 9*%*–4*%*).

For the question related to the level of education, 207 (99%) of 209 patients answered. Of these, six subjects (3%) stated that they had a primary school education (elementary school diploma), 41 subjects (20%) had a lower secondary school diploma (middle school), 114 (55%) had a higher secondary school diploma (high school), 16 subjects (8%) had a bachelor′s degree, and finally, 30 (14%) subjects had a master′s degree. None of the subjects responded that they had a PhD or other education.

Regarding the question on the type of genetic test result, a total of 204 (98%) of 209 patients responded. Of these, 147 (72%) reported that they received a negative noninformative test, 30 (15%) a positive test, and 27 (13%) a noninformative test due to a VUS finding.

Finally, regarding the question on the year of testing (2023 vs. 2024), 208 (99.5%) out of 209 subjects responded. Of these, 164 (79%) took the test and received the response in 2023, whereas 44 (21%) in 2024.

Patients′ demographics are reported below in Table 3.

**Table 3:** Patients′ demographic characteristics.

Patients′ demographics	*N* = 209 (100*%*)
**Age**	*N* = 197/209 (94*%*)
Median age: 56 years (range 25–81)	
• < 50 years	63 (32%)
• > 70 years	14 (7%)
• 50–70 years	120 (61%)
**Gender**	*N* = 208 (99.5*%*)
• Female	192 (92%)
• Male	16 (8%)
**Type of tumor**	*N* = 207 (99*%*)
• Invasive breast cancer	157 (76%)
• Healthy subjects	15 (7%)
• Gastrointestinal neoplasms (*n* = 14.7*%*)	14 (7%)
• Gynecological cancer (*n* = 6.3*%*)	6 (3%)
• Urological cancer	6 (3%)
• Multiple neoplasms	9 (4%)
**Level of education**	*N* = 207 (99*%*)
• Primary school education (elementary school diploma)	6 (3%)
• Lower secondary school diploma (middle school)	41 (20%)
• Higher secondary school diploma (high school)	114 (55%)
• Bachelor’s degree,	16 (8%)
• Master’s degree	30 (14%)
**Type of genetic test result**	*N* = 204 (98*%*)
• Negative noninformative test	147 (72%)
• Positive test	30 (15%)
• Noninformative test (VUS finding)	27 (13%)
**Year of testing (2023 vs. 2024)**	*N* = 208 (99.5*%*)
• 2023	164 (79%)
• 2024	44 (21%)

### 3.3. Distribution of Responses Based on GOS Questionnaire Items

The responses of subjects included in the study relating to the GOS questionnaire are reported in the table below (Table 4).

**Table 4:** Responses of subjects included in the study relating to the GOS questionnaire.

Genomics Outcome Scale	Total of responses n (%)	Strongly disagree *n* (%)	Disagree *n* (%)	Neither agree nor sisagree *n* (%)	Agree *n* (%)	Strongly agree *n* (%)
**Question 1**I can explain what the condition means to people outside family who may need to know.	209/209 (100%)	3 (2%)	6 (3%)	21 (10%)	55 (26%)	124 (59%)
**Question 2**I know who else in my family might be at risk for this condition.	209/209 (100%)	4 (2%)	3 (1%)	7 (3%)	20 (10%)	175 (84%)
**Question 3**When I think about the condition in my family, I get upset.	208/209 (99.5%)	12 (6%)	29 (14%)	32 (15%)	53 (26%)	82 (39%)
**Question 4**I know what I can do to change how this condition affects me/my children.	207/209 (99%)	11 (5%)	5 (2%)	33 (16%)	51 (25%)	107 (52%)
**Question 5**I can make decisions about the condition that might change my future or my child(ren)′s future.	207/209 (99%)	8 (4%)	9 (4%)	21 (10%)	47 (23%)	122 (59%)
**Question 6**I am able to make plans for the future.	208/209 (99.5%)	15 (7%)	14 (7%)	35 (17%)	31 (15%)	113 (54%)

### 3.4. Association Analysis Between Responses and Clinical Factors

#### 3.4.1. Age

Two age cutoffs were chosen: 50 years and 70 years. Globally, 63 patients were younger than 50 years and 14 were older than 70 years.

The analyses between the subgroups of patients younger than or older than 50 years are reported below.

**Figure 2:**
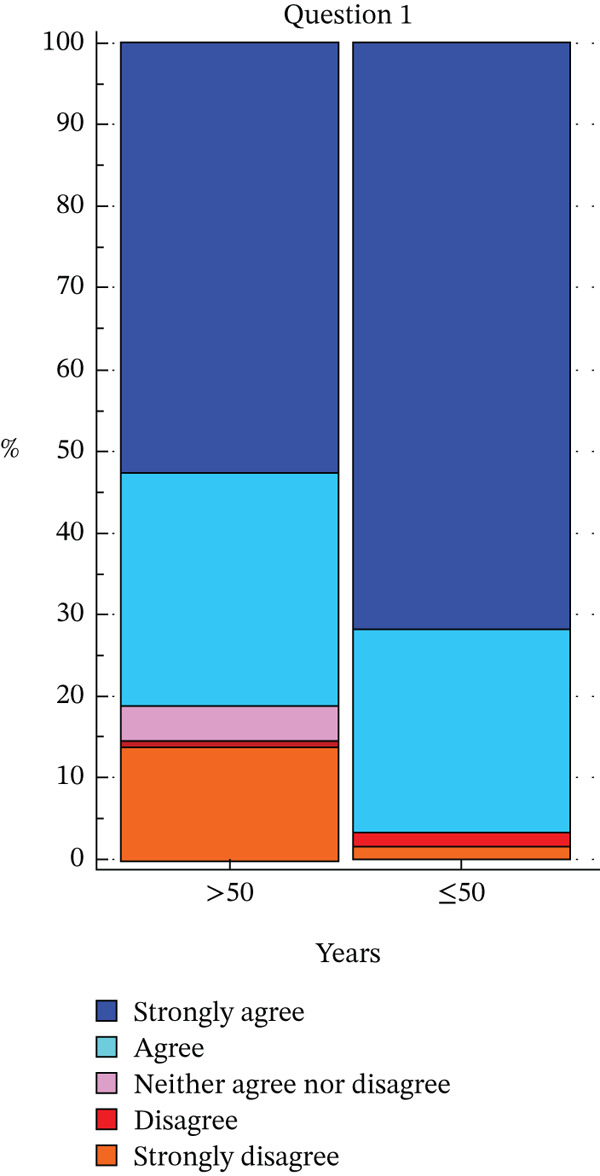
Distribution of responses to Question 1 (“I can explain what the condition means to people outside family who may need to know.”) according to age ≥ 50.

Regarding the answer to Question 1, a statistically significant difference was observed: people younger than 50 years answered *strongly agree* more often than people older than 50 years, whereas people older than 50 years answered *disagree* or *neither agree nor disagree* more often than those younger than 50 years (*p* = 0.0181) (Figure 2).

No other statistically significant differences were observed regarding Questions 2, 3, 4, 5, and 6 regarding subgroups of subjects considering cutoff of 50 years.

Moreover, no other significant differences for all the GOS questions were observed considering age cutoff of 70 years (Table 5). The complete results of the analysis can be found in Table S1.

**Table 5:** Chi‐square test results of association analyses between GOS questionnaire responses and stratification factors.

Genomics Outcome Scale (GOS) questions	Age (cutoff 50)	Age (cutoff 70)	Education level	Tumor type	Genetic testing result	Year of execution (2023 vs. 2024)
**Question 1**“I can explain what the condition means to people outside family who may need to know.”	**p** = 0.0181	*p* = 0.8397	**p** = 0.0028	*p* = 0.3383	*p* = 0.2570	*p* = 0.8706
**Question 2**“I know who else in my family might be at risk for this condition.”	*p* = 0.2355	*p* = 0.8778	*p* = 0.1171	*p* = 0.1662	**p** = 0.0175	*p* = 0.7222
**Question 3**“When I think about the condition in my family, I get upset.”	*p* = 0.4957	*p* = 0.5289	**p** = 0.0392	**p** = 0.0128	*p* = 0.2705	*p* = 0.7670
**Question 4**“I know what I can do to change how this condition affects me/my children.”	*p* = 0.7564	*p* = 0.4014	*p* = 0.2386	*p* = 0.1458	*p* = 0.7398	*p* = 0.5923
**Question 5**“I can make decisions about the condition that might change my future or my child(ren)′s future.”	*p* = 0.6629	*p* = 0.3795	**p** = 0.0287	**p** = 0.0307	**p** = 0.0090	*p* = 0.9907
**Question 6**“I am able to make plans for the future.”	*p* = 0.3023	*p* = 0.2476	*p* = 0.6684	*p* = 0.2099	*p* = 0.6021	*p* = 0.7038

*Note:* Bold texts refers to statistically significant items (statistically significant p).

#### 3.4.2. Education Level

Regarding the answer to Question 1, a statistically significant difference was observed based on the level of education (*p* = 0.0028). In particular, albeit the most frequent answer that was given from each group was *strongly agree*, this was given only by half of the population of patients who had primary school degree compared with 87% people with a master′s or bachelor′s degree.

Also regarding the answer to Question 3, a statistically significant difference was observed based on the level of education (*p* = 0.0392). People who answered *strongly agree* were mainly those with an elementary school or middle school diploma, whereas those who answered *strongly agree* less frequently were those with a master′s degree.

Regarding the answer to Question 5, a statistically significant difference was observed (*p* = 0.0287): people with an elementary school diploma answered *strongly disagree* mainly. None with a bachelor′s or master′s degree answered *strongly disagree*.

**Figure 3:**
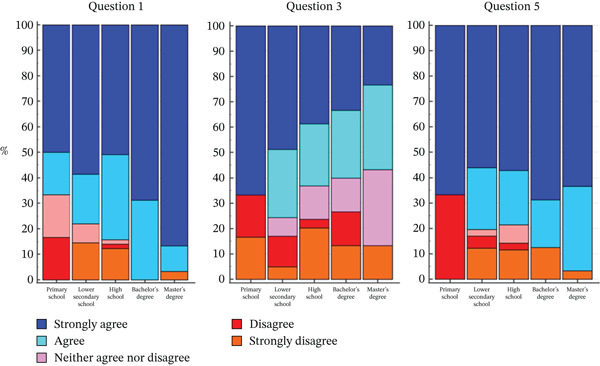
Distribution of responses to Questions 1 (“I can explain what the condition means to people outside family who may need to know.), 3 (“When I think about the condition in my family, I get upset,”), and 5 (“I can make decisions about the condition that might change my future or my child (ren)′s future.”) according to level of education.

Finally, no significant differences were observed on Questions 2, 4, and 6 regarding education level (Figure 3, Table 5). The complete results of the analysis can be found in Table S2.

#### 3.4.3. Type of Tumor

For the sake of statistical analyses, tumor types were categorized in the following subgroups: breast cancer, gynecological cancer (ovary, endometrium), urological tumor (prostate and urothelium), gastrointestinal cancer (including colorectal, pancreatic, and gastric cancer), multiple tumors, and nonaffected subjects.

No significant differences were observed on Questions 1, 2, 4, and 6 based on tumor type.

Regarding the answer to Question 3, a statistically significant difference was observed based on the type of tumor (*p* = 0.0128). Specifically, nonaffected subjects responded less frequently to *strongly agree*, whereas subjects with gastrointestinal cancers responded more frequently to *strongly agree*, followed by those with multiple tumors.

**Figure 4:**
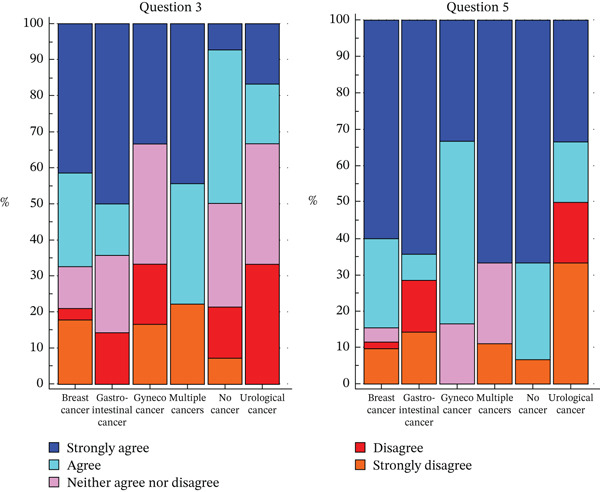
Distribution of responses to Questions 3 (“When I think about the condition in my family, I get upset.”) and 5 (“I can make decisions about the condition that might change my future or my child (ren)′s future.”) according to tumor type.

Moreover, regarding the answer to Question 5, a statistically significant difference was observed based on tumor type (*p* = 0.0307). In particular, patients with GI and urological cancer gave the most mixed results to Question 5 compared with other tumor types, as shown in Figure 4. (Figure 4, Table 5).) The complete results of the analysis can be found in Table S3.

#### 3.4.4. Genetic Testing Results

The questionnaire included the following possible answers: negative noninformative result (no pathogenic variants detected), positive result (pathogenic mutation found), noninformative result due to the presence of a VUS.

No significant differences were observed on Questions 1, 3, 4, and 6 regarding test result.

Regarding the answer to Question 2, a statistically significant difference was observed on test result (*p* = 0.0175). Patients carrying a VUS answered more often with a *strongly disagree*, whereas none of the patients with a positive test answered *strongly disagree* or *disagree*.

**Figure 5:**
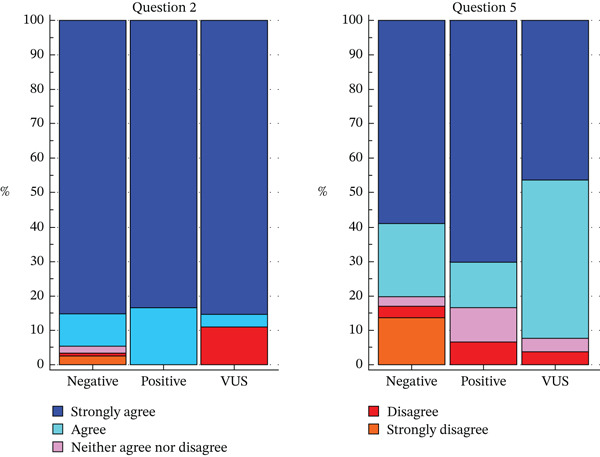
Distribution of responses to Questions 2(“I know who else in my family might be at risk for this condition.”) and 5 (“I can make decisions about the condition that might change my future or my child (ren)′s future.”) according to genetic testing result.

Regarding the answer to Question 5, also, a statistically significant difference was observed (*p* = 0.0090): patients carrying a VUS responded less frequently with *strongly agree*, whereas patients with a positive test responded more frequently with *strongly agree* (Figure 5, Table 5). The complete results of the analysis can be found in Table S4.

#### 3.4.5. Year of Test Execution

No significant differences were observed among Questions 1, 2, 3, 4, 5, and 6 regarding the year of test execution (2023 vs. 2024) (Table 5). The complete results of the analysis can be found in Table S5.

## 4. Discussion

In recent years, a widely increased genetic testing use has been reported, both for prevention and therapeutic purposes; this is partly due to the widespread use of biological drugs targeting specific molecular pathways (such as pathogenic variant affecting BRCA1 and BRCA2 genes). This process, called “mainstreaming genetic testing”, led to the use of genetic tests that are usually used to assess cancer predisposition also outside traditional genetics services and clinics [24].

However, despite the increasing use of genetic counseling in oncology, to date no validated assessment tools for evaluate quality of service offered by genetics centers have been used, either at European countries or in Italy, in particularly evaluating the OGC path. For this reason, the aim of the present study was to evaluate the effectiveness of OGC process at Our Center, using an easy‐to‐use, not “burdensome”, and easily interpretable assessment tool. For this purpose, we decided to use an Italian‐translated version of the GOS [25], which represents a faster and simplified version of GCOS‐24, another questionnaire that has been previously used in the field of clinical genetics services [20]. GOS and GCOS‐24 have been compared and the former has been demonstrated to be able to retain the ability to capture the theoretical construct of patient “empowerment” as the latter, despite having only six items compared with the 24 items of the GCOS‐24. [25]. Our objective was to assess OGC posttest phase effectiveness in terms of received information, clinical benefit, and acceptance of surveillance path based on the outcome of the test, patient and family awareness, and psychological impact.

Another relevant factor that posed the need for better understanding the perceived results of OGC posttest phase was also the fact that since June 2023, it has been introduced in our clinical practice as a unique multigene panel with NGS technology that does not simply include BRCA1 and BRCA2 or LS genes, but also several genes related to the HBOC and LS syndrome (BRCA1, BRCA2, PALB2, CHEK2, TP53, ATM, RAD50, RAD51C, RAD51D, MLH1, MSH2, MSH6, PMS2, and EPCAM). These genes are simultaneously analyzed in order to reduce referencing times, costs and, above all, to increase the test sensitivity to detect pathogenic variants, but it has led to greater test interpretation complexity, due to the increase in the rate of VUS detection. Wider gene panel testing has also led to “incidental findings”, as in the discovery of the presence of a pathogenic variant in those families that underwent testing even when family history and other criteria led to a pretest low probability of discovering a pathogenic variant.

In addition to the GOS questionnaire, also some clinical information such as age, gender, type of cancer, test result, and year of execution were collected, useful to highlight any differences in responses.

The population of 209 subjects included in the study consists mainly of female subjects (92%) affected predominantly by breast cancer (76%). Based on different cancer types prevalence it is not surprising that the most frequent test proposed and performed at Our Center is for HBOC Syndrome, including BRCA1 and BRCA2 genes. The median age of subjects who responded to the questionnaire is 56 years old, but the very wide age range (25–81) suggests that the proposed compilation methods (by telephone or by e‐mail compilation) should be easily accessible by different age groups. We cannot fully prove this fact since the test was conducted anonymously and thus we could not perform an analysis based on the rate of questionnaire completion and age or other clinical factors. We aim in the future to assess whether such differences in completion rate do exist. We also aim to assess whether differences in the answers would be detected by offering the GOS questionnaire before and after the OGC.

Regarding education level, most patients reported having a high school diploma (55 %), followed by a lower middle school diploma (20 %), whereas only 22 % had a bachelor′s degree. Most of the enrolled subjects received a negative noninformative test (72 %), wheras only 15 % of them carried a pathogenic variant or a VUS.

We also analyzed in detail the answers given by the participants to the GOS questionnaire.

Regarding Question 1 “I can explain what the condition means to people outside family who may need to know”, most patients (85%) answered affirmatively, thus confirming the OGC usefulness in providing understandable information. In particular, subjects who answered with a higher probability *strongly agree* are those aged less than or equal to 50 years and holding a degree (three‐year or master′s degree). On the other hand, subjects with a lower level of education (primary school certificate) responded more frequently in a negative way. These differences were statistically significant and may suggest how the complexity of information contained within OGC can be better understood by people with higher levels of education. Also, younger people would seem to have tools for a better understanding of test results than older people. In our opinion, a possible explanation could be due to greater knowledge of concerns related to hereditary cancers thanks to the dissemination of information by the “mass media” in younger people. This may lead to a reflection on how the test is reported and explained to people who, by level of education or age, might not be able to completely understand the results when explained in the conventionally used ways.

Analyzing Question 2 “I know who else in my family might be at risk for this condition” answers, almost all subjects (94%) answered *strongly agree* or *agree*, whereas only four people in total (2%) answered *strongly disagree*. A good awareness of which other relatives may be at risk was found to be cross‐sectional and independent of other factors such as age, gender, level of education, and type of cancer diagnosis, whereas an interesting finding emerged when answers were correlated with genetic test results: in particular, subjects carrying a VUS answered more frequently *strongly disagree*, unlike subjects carrying a pathogenic variant, where none answered negatively. This statistically significant difference highlights the great interpretative problem of VUS leading to the difficulty of transmitting information in a clear and understandable way and the patients′ perceived “lack” of solid information leading to psychological problems.

The following Question 3 “When I think about the condition in my family, I get upset” elicited interesting answers: in fact about two‐thirds (65%) of examined subjects replied that they were somewhat concerned about their condition, whereas only 20% replied that they were generally unconcerned. From subgroup analyses, subjects that expressed the greatest concern were those with lowest level of education (primary or lower secondary school certificate) and patients with gastrointestinal neoplasm or multiple neoplasms. Understandably, those not affected by cancer expressed a lower level of concern. We believe that the response to this specific item of the questionnaire might have been more influenced by the prognostic implications of the neoplasm from which the compilers were affected rather than the prognostic implications of genetic testing: in particular, within the subgroup “gastrointestinal tumors” there were several cases of pancreatic cancer, which usually have an unfavorable prognosis. The worse prognosis of pancreatic cancer compared to other tumor types is also a well‐known fact to the general populace, thus leading to increased fear and worry in subjects that were diagnosed with this disease [32, 33].

In Question 4 “I know what I can do to change how this condition affects me/my children” that measures the degree of understanding of proposed surveillance procedures for theirself and their family, most subjects (77%) responded declaring full (or almost) awareness, independent of all clinical factors, emphasizing the important role of OGC in the process of patient empowerment. To note, none of the clinical factors we used to identify differences in questionnaire responses appear to have identified groups of patients in which OGC was not able to increase the empowerment of those who receive the test results.

Regarding Question 5 “I can make decisions about the condition that might change my future or my child (ren)′s future”, as for the previous question, most subjects (72%) declared that they were able, completely or partially, to make decisions to change their own future or that of their family members, regardless of age. Significant differences emerged based on the level of education, the type of neoplastic pathology and the test result. In fact, subjects with the lowest level of education and carriers of a VUS were found to be less able to make decisions for themselves and their family members, whereas an interesting fact is that subjects carriers of a pathogenic variant are those who most frequently answered affirmatively, demonstrating that the OGC represents a decisive and irreplaceable moment in carrier patients in terms of empowerment, to plan appropriate surveillance, screening and prevention measures. Subjects carrying a pathogenic variant have well‐codified surveillance protocols available and/or with limited margins of uncertainty regarding their effectiveness, and this can explain the high percentage of affirmative responses. Other previous papers also seem to suggest that miscomprehension of VUS is common by counselees, particularly in those with lower education, highlighting the importance of VUS‐related educational interventions for both VUS carriers and their referring physicians [34, 35].

Interestingly, a significant difference also emerged based on type of cancer diagnosis, without being able to highlight differences that can be categorized based on the histological type: it cannot be excluded that could be partly due to the intrinsic prognosis of the various types of tumor and to other factors related to the neoplastic pathology, such as the stage of the disease, which were not collected in our questionnaire.

Finally, Question 6 “I am able to make plans for the future”, which focuses on the impact of the information acquired during genetic counseling on individual sphere and on planning one′s life, 69 % of subjects answered completely or almost completely affirmatively, 17% adopted a neutral position of uncertainty whereas overall 14% of the subjects answered partially or completely negatively. It is interesting to note that, in contrast to what was expected, no differences emerged based on clinical stratification factors examined.

Overall, from data collected with the GOS questionnaire, it seems to emerge that the OGC path represents a useful and effective tool to make the patient informed about his/her condition and to provide useful tools in terms of surveillance and prevention, also in order to plan conscious choices to his/her future and that of his/her family members

The limitations of this study lie in sample size, which albeit quite large, can further be increased in the future. We also aim to specifically increase the number of subjects that are currently underrepresented in this analysis because of the smaller number of subjects, such as those who are not affected by breast cancer and who are not females. Finally, the starting sample presents a lack of homogeneity in terms of diagnostic suspicion, as it included both patients enrolled for HBOC tests and for LS.

Unfortunately, in Italy and Europe there are no similar experiences in the field of OGC of hereditary‐familial tumors: a single work presented in the form of an abstract at the XXX National IMI Congress (Italian Melanoma Intergroup) in September 2024 obtained similar results to those of the present study, highlighting the usefulness of genetic telecounseling in a smaller sample (176 subjects) of patients undergoing genetic testing for familial melanoma, always using the GOS questionnaire [36].

## 5. Conclusions

In conclusion, the administration of a validated questionnaire for evaluation of the OGC pathway was feasible, with a high patients′ acceptance rate. This allowed proper assessment of multigene panel test effectiveness. In the future, it would be interesting to increase sample size to highlight any differences and identify any critical points in the process, in order to develop improvement and correction actions, where necessary.

**Nomenclature**
OGC: oncological genetic counseling
GOS: Genomics Outcome Scale
HRQoL: health‐related quality of life
PROMs: patient‐reported outcome measures
GCOS‐24: Genetic Counseling Outcome Scale.
AOU: Azienda Ospedaliero Universitaria
HBOC: hereditary breast/ovarian cancer
LS: Lynch syndrome
NGS: next generation sequencing
MLPA: multiplex ligation probe amplification
ENIGMA: evidence‐based network for the interpretation of germline mutant allele
ACMG: American College of Medical Genetics and Genomics
InSiGHT: International Society for Gastrointestinal Hereditary Tumors
IARC: International Agency for Research on Cancer.
VUS: variant of unknown significance
IMI: Italian Melanoma Intergroup

## Author Contributions

Conceptualization: E.M., R.C (Rebecca Chiariotti)., R.G., and R.B (Rossana Berardi); metodology: E.M. and R.G.; software: R.G.; validation: E.M., R.G., and R.B. (Rossana Berardi); formal analysis: R.G.; investigation ad data collection: E.M., R.C. (Rebecca Chiariotti), F.B., C.B., L.B, E.A., N.C., E.N.S. F.M., V.A., G.M., C.C., A.M., C.D.F., E.B., R.B. (Raffaella Bracci), R.C. (Rita Chiari), M.D.P., R.B. (Renato Bisonni), L.F., N.B., R.R.S.; resources: R.B. (Rossana Berardi); data curation: E.M., R.C. (Rebecca Chiariotti), R.G.; writing and original draft preparation: E.M, R.C. (Rebecca Chiariotti), R.G., and R.B. (Rossana Berardi); writing and review and editing: R.G. and R.B. (Rossana Berardi).

## Funding

No funding was received for this manuscript.

## Disclosure

All authors have read and agreed to the published version of the manuscript.

## Ethics Statement

The study did not require ethical approval.

## Consent

Written informed consent was obtained from all subjects involved in the study.

## Conflicts of Interest

The authors declare no conflicts of interest.

## Supporting information


**Supporting Information** Additional supporting information can be found online in the Supporting Information section. (Supporting information) Table S1: Complete results of GOS questions stratified by different age groups (< or > 50 years old, < or > 70 years old or age not known). Table S2: Complete results of GOS questions stratified by different levels of instruction. Table S3: Complete results of GOS questions stratified by different sites of tumor involvement. Table S4: Complete results of GOS questions stratified by gene test results. Table S5: Complete results of GOS questions stratified by year of testing.

## Data Availability

All available data can be provided by the corresponding author on demand.
