# Supplementary material for: Oncological Genetic Counseling in Hereditary Breast and Ovarian Cancers and Lynch Syndrome High‐Risk Subjects: Evaluation of Efficacy and Outcomes Using the Genomics Outcome Scale
Source: Hum Mutat. 2026 Mar 9;2026:7754087. doi: 10.1155/humu/7754087 (PMC12968728; doi:10.1155/humu/7754087)
Supplement: Supplementary file 1 — Supporting Information Additional supporting information can be found online in the Supporting Information section. (Supporting information) Table S1: Complete results of GOS questions stratified by different age groups (< or > 50 years old, < or > 70 years old or age not known). Table S2: Complete results of GOS questions stratified by different levels of instruction. Table S3: Complete results of GOS questions stratified by different sites of tumor involvement. Table S4: Complete results of GOS questions stratified by gene test results. Table S5: Complete results of GOS questions stratified by year of testing. [file HUMU-2026-7754087-s001.docx]

**Supplementary Description**

| **Genomics Outcome Scale** | **Total of responses**  **n (%)** | **Strongly Disagree**  **n (%)** | **Disagree**  **n (%)** | **Neither Agree nor Disagree**  **n (%)** | **Agree**  **n (%)** | **Strongly Agree**  **n (%)** |
| --- | --- | --- | --- | --- | --- | --- |
| **“I can explain what the condition means to people outside family who may need to know”** | <70: 183  >70: 14  <50:60  >50: 137  Age not known: 12 | 2 (1%)  0 (0%)  1 (2%)  1 (1%)  1 (8%) | 5 (3%)  1 (7%)  0 (0%)  6 (4%)  0 (0%) | 18 (10%)  2 (14%)  1 (2%)  19 (14%)  0 (0%) | 50 (27%)  4 (28%)  15 (25%)  39 (28%)  1 (8%) | 108 (59%)  7 (50%)  43 (72%)  72 (52%)  10 (83%) |
| **“I know who else in my family might be at risk for this condition”** | <70:183  >70:14  <50:60  >50: 137  Age not known:12 | 2 (1%)  0 (0%)  0 (0%)  2 (1%)  2 (17%) | 3 (2%)  0 (0%)  0 (0%)  3 (2%)  0 (0%) | 7 (4%)  0 (0%)  4 (7%)  3 (2%)  0 (0%) | 19 (10%)  1 (8%)  8 (13%)  12 (9%)  0 (0%) | 152 (83%)  13 (92%)  48 (80%)  117 (85%)  10 (83%) |
| **“When I think about the condition in my family, I get upset”** | <70:182  >70:14  <50:59  >50:137  Age not known:12 | 11 (6%)  1 (7%)  5 (8%)  7 (5%)  0 (0%) | 26 (14%)  1 (7%)  9 (15%)  18 (13%)  2 (17%) | 26 (14%)  4 (28%)  7 (12%)  23 (17%)  2 (17%) | 50 (27%)  2 (14%)  19 (33%)  33 (24%)  1 (8%) | 69 (38%)  6 (42%)  19 (33%)  56 (41%)  7 (58%) |
| **“I know what I can do to change how this condition affects me/my children”** | <70:181  >70:14  <50:60  >50:135  Age not known:12 | 7 (4%)  0 (0%)  1 (2%)  6 (4%)  4 (33%) | 5 (3%)  0 (0%)  1 (2%)  4 (3%)  0 (0%) | 30 (16%)  3 (21%)  11 (18%)  22 (16%)  0 (0%) | 47 (26%)  1 (7%)  13 (22%)  35 (26%)  3 (25%) | 92 (51%)  10 (71%)  34 (57%)  68 (50%)  5 (42%) |
| **“I can make decisions about the condition that might change my future or my child(ren)’s future”** | <70:181  >70:14  <50:60  >50:135  Age not known:12 | 5 (3%)  0 (0%)  1 (2%)  4 (3%)  3 (25%) | 7 (4%)  2 (14%)  1 (2%)  8 (4%)  0 (0%) | 18 (10%)  2 (14%)  6 (10%)  14 (10%)  1 (8%) | 43 (24%)  2 (14%)  16 (27%)  29 (21%)  2 (17%) | 108 (60%)  8 (57%)  36 (60%)  80 (59%)  6 (50%) |
| **“I am able to make plans for the future”** | <70:182  >70:14  <50:60  >50:136  Age not known:12 | 10 (5%)  3 (21%)  3 (5%)  10 (7%)  2 (17%) | 13 (7%)  1 (7%)  2 (3%)  12 (9%)  0 (0%) | 32 (17%)  2 (14%)  9 (15%)  25 (18%)  1 (8%) | 27 (15%)  2 (14%)  7 (12%)  22 (16%)  2 (17%) | 100 (55%)  6 (43%)  39 (65%)  67 (49%)  7 (58%) |

**Supplementary Table 1:** Complete results of GOS questions stratified by different age groups (< or >50 years old, < or >70 years old or age not known).

| **Genomics Outcome Scale** | **Total of responses**  **n (%)** | **Strongly Disagree**  **n (%)** | **Disagree**  **n (%)** | **Neither Agree nor Disagree**  **n (%)** | **Agree**  **n (%)** | **Strongly Agree**  **n (%)** |
| --- | --- | --- | --- | --- | --- | --- |
| “I can explain what the condition means to people outside family who may need to know” | Primary School:6  Lower Secondary:41  Higher Secondary:114  Bachelor’s Degree:16  Master Degree:30 | 1 (17%)  0 (0%)  2 (2%)  0 (0%)  0 (0%) | 1 (17%)  3 (7%)  2 (2%)  0 (0%)  0 (0%) | 0 (0%)  6 (14%)  14 (12%)  0 (0%)  1 (3%) | 1 (17%)  8 (19%)  38 (33%)  5 (31%)  3 (10%) | 3 (50%)  24 (58%)  58 (51%)  11 (69%)  26 (87%) |
| “I know who else in my family might be at risk for this condition” | Primary School:6  Lower Secondary:41  Higher Secondary:114  Bachelor Degree:16  Master Degree:30 | 0 (0%)  0 (0%)  1 (1%)  0 (0%)  2 (7%) | 1 (17%)  0 (0%)  2 (2%)  0 (0%)  0 (0%) | 0 (0%)  1 (2%)  4 (4%)  1 (6%)  0 (0%) | 0 (0%)  6 (15%)  12 (10%)  1 (6%)  1 (3%) | 5 (83%)  34 (83%)  95 (83%)  14 (88%)  27 (90%) |
| “When I think about the condition in my family, I get upset” | Primary School:6  Lower Secondary:41  Higher Secondary:114  Bachelor Degree:15  Master Degree:30 | 1 (17%)  5 (12%)  4 (3%)  2 (13%)  0 (0%) | 0 (0%)  3 (7%)  15 (13%)  2 (13%)  9 (30%) | 1 (17%)  2 (5%)  23 (20%)  2 (13%)  4 (13%) | 0 (0%)  11 (27%)  28 (25%)  4 (27%)  10 (33%) | 4 (67%)  20 (49%)  44 (39%)  5 (33%)  7 (23%) |
| “I know what I can do to change how this condition affects me/my children” | Primary School:6  Lower Secondary:41  Higher Secondary:112  Bachelor Degree:16  Master Degree:30 | 2 (33%)  3 (7%)  5 (4%)  0 (0%)  0 (0%) | 0 (0%)  1 (2%)  4 (4%)  0 (0%)  0 (0%) | 1 (17%)  7 (17%)  19 (17%)  1 (6%)  5 (17%) | 0 (0%)  10 (24%)  30 (27%)  4 (25%)  6 (20%) | 3 (50%)  20 (49%)  54 (48%)  11 (69%)  19 (63%) |
| “I can make decisions about the condition that might change my future or my child(ren)’s future” | Primary School:6  Lower Secondary:41  Higher Secondary:112  Bachelor Degree:16  Master Degree:30 | 2 (33%)  2 (5%)  3 (3%)  0 (0%)  0 (0%) | 0 (0%)  1 (2%)  8 (7%)  0 (0%)  0 (0%) | 0 (0%)  5 (12%)  13 (12%)  2 (12%)  1 (3%) | 0 (0%)  10 (24%)  24 (21%)  3 (19%)  10 (33%) | 4 (67%)  23 (56%)  64 (57%)  11 (69%)  19 (63%) |
| “I am able to make plans for the future” | Primary School:6  Lower Secondary:41  Higher Secondary:113  Bachelor Degree:16  Master Degree:30 | 2 (33%)  5 (12%)  6 (5%)  1 (12%)  1 (3%) | 0 (0%)  3 (7%)  10 (9%)  0 (0%)  1 (3%) | 1 (17%)  5 (12%)  21 (18%)  3 (19%)  5 (17%) | 1 (17%)  6 (15%)  17 (15%)  2 (12%)  4 (13%) | 2 (33%)  22 (54%)  59 (52%)  10 (63%)  19 (63%) |

**Supplementary Table 2:** Complete results of GOS questions stratified by different level of instruction.

| **Genomics Outcome Scale** | **Total of responses**  **n (%)** | **Strongly Disagree**  **n (%)** | **Disagree**  **n (%)** | **Neither Agree nor Disagree**  **n (%)** | **Agree**  **n (%)** | **Strongly Agree**  **n (%)** |
| --- | --- | --- | --- | --- | --- | --- |
| “I can explain what the condition means to people outside family who may need to know” | Breast Cancer:157  GI Cancer:14  Gynecological Cancer:6  Urological Cancer:6  Multiple Cancer:9  No Cancer:15 | 2 (1%)  0 (0%)  0 (0%)  0 (0%)  0 (0%)  0 (0%) | 4 (2%)  0 (0%)  0 (0%)  1 (17%)  1 (11%)  0 (0%) | 14 (9%)  1 (7%)  3 (50%)  1 (17%)  1 (11%)  1 (7%) | 42 (27%)  3 (21%)  0 (0%)  1 (17%)  3 (33%)  6 (40%) | 95 (60%)  10 (71%)  3 (50%)  3 (50%)  4 (44%)  8 (53%) |
| “I know who else in my family might be at risk for this condition” | Breast Cancer:157  GI Cancer:14  Gynecological Cancer:6  Urological Cancer:6  Multiple Cancer:9  No Cancer:15 | 3 (2%)  0 (0%)  0 (0%)  0 (0%)  0 (0%)  0 (0%) | 1 (1%)  0 (0%)  0 (0%)  1 (17%)  1 (11%)  0 (0%) | 6 (4%)  0 (0%)  0 (0%)  1 (17%)  0 (0%)  0 (0%) | 15 (10%)  2 (14%)  1 (17%)  1 (17%)  1 (11%)  0 (0%) | 132 (84%)  12 (86%)  5 (83%)  3 (50%)  7 (78%)  15 (100%) |
| “When I think about the condition in my family, I get upset” | Breast Cancer:157  GI Cancer:14  Gynecological Cancer:6  Urological Cancer:6  Multiple Cancer:9  No Cancer:15 | 5 (3%)  2 (14%)  1 (17%)  2 (33%)  0 (0%)  2 (13%) | 18 (11%)  3 (21%)  2 (33%)  2 (33%)  0 (0%)  4 (27%) | 28 (18%)  0 (0%)  1 (17%)  0 (0%)  2 (22%)  1 (7%) | 41 (26%)  2 (14%)  0 (0%)  1 (17%)  3 (33%)  6 (40%) | 65 (41%)  7 (50%)  2 (33%)  1 (17%)  4 (44%)  1 (7%) |
| “I know what I can do to change how this condition affects me/my children” | Breast Cancer:155  GI Cancer:14  Gynecological Cancer:6  Urological Cancer:6  Multiple Cancer:9  No Cancer:15 | 6 (4%)  2 (14%)  0 (0%)  1 (17%)  0 (0%)  0 (0%) | 5 (3%)  0 (0%)  0 (0%)  0 (0%)  0 (0%)  0 (0%) | 26 (17%)  0 (0%)  0 (0%)  2 (33%)  2 (22%)  3 (20%) | 35 (22%)  2 (14%)  5 (83%)  2 (33%)  3 (33%)  4 (27%) | 83 (54%)  10 (71%)  1 (17%)  1 (17%)  4 (44%)  8 (53%) |
| “I can make decisions about the condition that might change my future or my child(ren)’s future” | Breast Cancer:155  GI Cancer:14  Gynecological Cancer:6  Urological Cancer:6  Multiple Cancer:9  No Cancer:15 | 3 (2%)  2 (14%)  0 (0%)  1 (17%)  0 (0%)  0 (0%) | 6 (4%)  0 (0%)  1 (17%)  0 (0%)  2 (22%)  0 (0%) | 15 (10%)  2 (14%)  0 (0%)  2 (33%)  1 (11%)  1 (7%) | 38 (24%)  1 (7%)  3 (50%)  1 (17%)  0 (0%)  4 (27%) | 93 (60%)  9 (64%)  2 (33%)  2 (33%)  6 (67%)  10 (67%) |
| “I am able to make plans for the future” | Breast Cancer:156  GI Cancer:14  Gynecological Cancer:6  Urological Cancer:6  Multiple Cancer:9  No Cancer:15 | 10 (6%)  1 (7%)  0 (0%)  1 (17%)  2 (22%)  0 (0%) | 11 (7%)  2 (14%)  0 (0%)  0 (0%)  1 (11%)  0 (0%) | 31 (20%)  2 (14%)  1 (17%)  0 (0%)  1 (11%)  0 (0%) | 25 (16%)  0 (0%)  1 (17%)  0 (0%)  3 (33%)  2 (13%) | 79 (51%)  9 (64%)  4 (67%)  5 (83%)  2 (22%)  13 (87%) |

**Supplementary Table 3:** Complete results of GOS questions stratified by different site of tumor involvement.

| **Genomics Outcome Scale** | **Total of responses**  **n (%)** | **Strongly Disagree**  **n (%)** | **Disagree**  **n (%)** | **Neither Agree nor Disagree**  **n (%)** | **Agree**  **n (%)** | **Strongly Agree**  **n (%)** |
| --- | --- | --- | --- | --- | --- | --- |
| “I can explain what the condition means to people outside family who may need to know” | Positive:30  Negative:147  VUS: 27 | 0 (0%)  3 (2%)  0 (0%) | 0 (0%)  6 (4%)  0 (0%) | 1 (3%)  14 (9%)  5 (18%) | 7 (23%)  38 (26%)  10 (37%) | 22 (73%)  86 (58%)  12 (44%) |
| “I know who else in my family might be at risk for this condition” | Positive:30  Negative:147  VUS: 27 | 0 (0%)  1 (1%)  3 (11%) | 0 (0%)  3 (2%)  0 (0%) | 0 (0%)  4 (3%)  0 (0%) | 5 (17%)  14 (10%)  1 (4%) | 25 (83%)  125 (85%)  23 (85%) |
| “When I think about the condition in my family, I get upset” | Positive:30  Negative:147  VUS: 27 | 3 (10%)  7 (5%)  1 (4%) | 3 (10%)  20 (14%)  6 (22%) | 4 (13%)  18 (12%)  7 (26%) | 7 (23%)  37 (25%)  8 (30%) | 13 (43%)  64 (43%)  5 (18%) |
| “I know what I can do to change how this condition affects me/my children” | Positive:30  Negative:145  VUS: 27 | 1 (3%)  8 (5%)  1 (4%) | 0 (0%)  4 (3%)  0 (0%) | 2 (7%)  27 (19%)  4 (15%) | 9 (30%)  34 (23%)  7 (26%) | 18 (60%)  72 (50%)  15 (56%) |
| “I can make decisions about the condition that might change my future or my child(ren)’s future” | Positive:30  Negative:146  VUS: 26 | 2 (7%)  5 (3%)  1 (4%) | 3 (10%)  4 (3%)  1 (4%) | 0 (0%)  20 (14%)  0 (0%) | 4 (13%)  31 (21%)  12 (46%) | 21 (70%)  86 (59%)  12 (46%) |
| “I am able to make plans for the future” | Positive:30  Negative:146  VUS: 27 | 3 (10%)  10 (7%)  1 (4%) | 1 (3%)  11 (8%)  2 (7%) | 3 (10%)  26 (18%)  5 (18%) | 3 (10%)  26 (18%)  2 (7%) | 20 (67%)  73 (50%)  17 (63%) |

**Supplementary Table 4:** Complete results of GOS questions stratified by gene test results.

| **Genomics Outcome Scale** | **Total of responses**  **n (%)** | **Strongly Disagree**  **n (%)** | **Disagree**  **n (%)** | **Neither Agree nor Disagree**  **n (%)** | **Agree**  **n (%)** | **Strongly Agree**  **n (%)** |
| --- | --- | --- | --- | --- | --- | --- |
| “I can explain what the condition means to people outside family who may need to know” | Year of test 2023:163  Year of test 2024:44 | 2 (1%)  1 (2%) | 5 (3%)  1 (2%) | 14 (9%)  6 (16%) | 43 (26%)  12 (27%) | 99 (61%)  24 (54%) |
| “I know who else in my family might be at risk for this condition” | Year of test 2023:163  Year of test 2024:44 | 2 (1%)  0 (0%) | 3 (2%)  1 (2%) | 5 (3%)  2 (4%) | 17 (10%)  3 (7%) | 136 (83%)  38 (86%) |
| “When I think about the condition in my family, I get upset” | Year of test 2023:162  Year of test 2024:44 | 7 (4%)  4 (9%) | 23 (14%)  6 (14%) | 26 (16%)  6 (14%) | 43 (26%)  10 (23%) | 63 (39%)  18 (41%) |
| “I know what I can do to change how this condition affects me/my children” | Year of test 2023:162  Year of test 2024:43 | 8 (5%)  3 (7%) | 3 (2%)  2 (5%) | 24 (15%)  8 (19%) | 39 (24%)  12 (28%) | 88 (54%)  18 (42%) |
| “I can make decisions about the condition that might change my future or my child(ren)’s future” | Year of test 2023:162  Year of test 2024:43 | 6 (4%)  2 (5%) | 7 (4%)  2 (5%) | 16 (10%)  5 (12%) | 36 (22%)  10 (23%) | 97 (60%)  24 (56%) |
| “I am able to make plans for the future” | Year of test 2023:163  Year of test 2024:43 | 10 (6%)  5 (12%) | 11 (7%)  3 (7%) | 27 (16%)  8 (19%) | 24 (15%)  7 (16%) | 91 (59%)  20 (46%) |

**Supplementary Table 5**: Complete results of GOS questions stratified by year of testing.
